# 

**Published:** 1887-10

**Authors:** 


CONTENTS:

ARTICLE I.—Ninth International Medical Congress ...... .................. 241
" II.—British Dental Association...... ........... ..................... 260
" III.—Dental Ethics. By T. H. Lipscomb ..... ............ .. .... 275
" IV.—The Abuse of Dental Charity..... ........................ .. 281

EDITORIAL, ETC.

North Carolina's Amended Dental Law ............................ ... ...... 285

OBITUARY.

John McCalla, D. D. S.......................................................... 287

BIBLIOGRAPHICAL.

The American System of Dentistry .......... ........................ .... ...... 287
Holden's Anatomy ... ....... ...... ........... ........... ......................... 288
Transactions of the Illinois State Dental Society..... ..... ................... 288

